# Individual differences in eye blink rate predict both transient and tonic pupil responses during reversal learning

**DOI:** 10.1371/journal.pone.0185665

**Published:** 2017-09-29

**Authors:** Joanne C. Van Slooten, Sara Jahfari, Tomas Knapen, Jan Theeuwes

**Affiliations:** Department of Applied and Experimental Psychology, Vrije Universiteit, Amsterdam, Noord-Holland, The Netherlands; Tokai University, JAPAN

## Abstract

The pupil response under constant illumination can be used as a marker of cognitive processes. In the past, pupillary responses have been studied in the context of arousal and decision-making. However, recent work involving Parkinson's patients suggested that pupillary responses are additionally affected by reward sensitivity. Here, we build on these findings by examining how pupil responses are modulated by reward and loss while participants (N = 30) performed a Pavlovian reversal learning task. In fast (transient) pupil responses, we observed arousal-based influences on pupil size both during the expectation of upcoming value and the evaluation of unexpected monetary outcomes. Importantly, after incorporating eye blink rate (EBR), a behavioral correlate of striatal dopamine levels, we observed that participants with lower EBR showed stronger pupil dilation during the expectation of upcoming reward. Subsequently, when reward expectations were violated, participants with lower EBR showed stronger pupil responses after experiencing unexpected loss. Across trials, the detection of a reward contingency reversal was reflected in a slow (tonic) dilatory pupil response observed already several trials prior to the behavioral report. Interestingly, EBR correlated positively with this tonic detection response, suggesting that variability in the arousal-based detection response may reflect individual differences in striatal dopaminergic tone. Our results provide evidence that a behavioral marker of baseline striatal dopamine level (EBR) can potentially be used to describe the differential effects of value-based learning in the arousal-based pupil response.

## Introduction

Pupil diameter fluctuations offer a non-invasive signal that reflects activity of neuromodulatory brain regions such as locus coeruleus [1,2] and superior colliculus [3]. Functionally, these pupil diameter fluctuations have been shown to track arousal-based influences on decision-making at multiple timescales [4–9]. Pupil dilations also track surprise caused by unexpected events [10–12], also when unexpected outcomes were tied to reward [13].

Recent studies with Parkinson’s patients have shown that patients’ pupil dilations to cues signaling monetary reward were reduced compared to age-matched controls. Crucially, dopaminergic medication reinstated these reward-anticipation pupil dilations [14,15], suggesting that pupil dilations may reflect changes in reward sensitivity, which in turn are likely related to changes in baseline dopamine levels in the striatum [16–19]. In the present study, we investigated in a healthy population the extent to which the expectation and evaluation of monetary outcomes affected pupil dilations and whether eye blink rate (EBR), a behavioral marker of striatal dopaminergic tone [20], was related to these pupil responses.

There is compelling evidence that EBR, or the frequency of blinks per unit time, is at least partly regulated by striatal dopamine levels [20–28], which in turn affects how individuals learn from reinforcing feedback [29]. Higher striatal dopamine levels facilitate the learning of positive outcomes [18,19], whereas lower striatal dopamine levels -observed in Parkinson’s disease- facilitate learning from negative outcomes [16,17]. Recently, EBR was found to predict participants’ value-based choice strategy in a reinforcement learning task [30,31], where lower EBR predicted a choice strategy focused on avoiding negative outcomes [31]. Consistently, pharmacologically decreasing striatal dopaminergic tone, as indexed by decreased EBR, led to increased punishment aversion in individuals with relatively high EBR prior to the pharmacological manipulation [30]. These studies suggest a relation between EBR and the outcome of a reinforcement learning processes. However, whether (and how) EBR relates to the instantaneous expectation or evaluation of value is currently unknown. Potentially, the simultaneous analysis of EBR and pupil responses during these types of events provide a non-invasive way to investigate value-based learning as it takes place.

**Fig 1:**
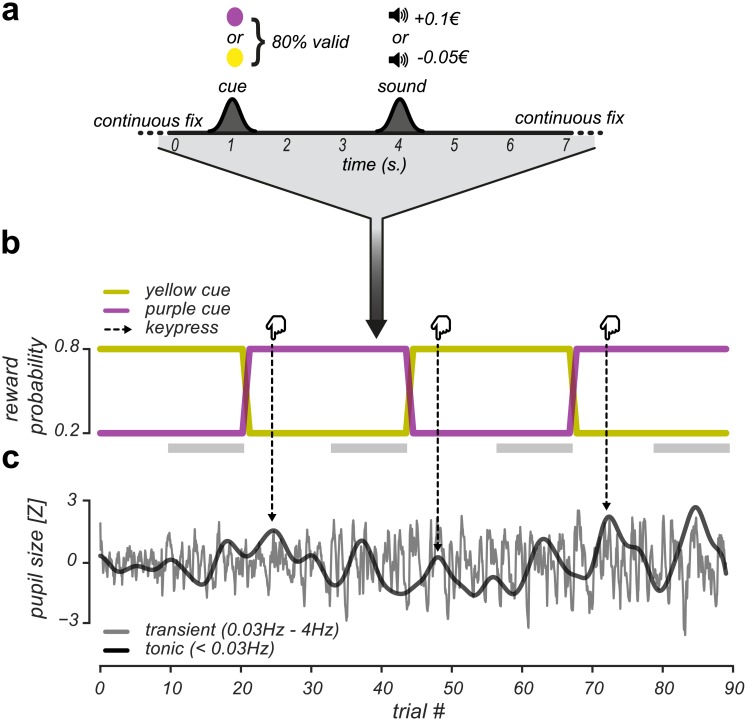
Pavlovian probabilistic reversal learning task and independent pupil signals. (A): Example trial. Participants continuously fixated a white dot at the center of the screen. After 500ms, the dot turned into a cue (either purple or yellow) that signaled for 1000ms a monetary outcome with 80% validity. After 3000ms, the monetary outcome (either reward or loss) was indicated by a sound. (B): Example run. The participant monitored the reward contingency across trials and reported a detected reversal in the reward contingency with a keypress. Yellow and purple lines indicate the reward probability of the cues, which reversed three times during this particular run. These reward contingency reversals constituted four reversal blocks: one block prior and 3 blocks after the experimental reversals. Dashed arrows indicate the moments in time the participant reported a detected reversal and highlight the correspondence between reversal detection and increases in tonic pupil size (see panel C). Grey bars below the cue reward probabilities indicate the latter half of trials within a reversal block that were used in the analyses of transient pupil responses. (C): Example of independently filtered tonic and transient pupil signals across a run.

To investigate whether pupil responses and EBR could be utilized to track value-based learning [32], we conducted a Pavlovian reversal learning experiment (Fig 1) in which probabilistic cues signaled upcoming positive or negative monetary outcomes. Our design elicited the learning of cue-outcome contingencies and triggered unexpected positive (U+) and unexpected negative (U-) events when value expectations were violated. At random intervals, cue-outcome contingencies were reversed and participants were asked to report these reversals. This task allowed us to separately study slow, tonic pupil responses during reversal detection across multiple trials [6] and fast, transient pupil responses during the expectation [33–38] and evaluation [13] of reward and loss. Moreover, we equated the sensory and monetary impact of positive and negative outcomes to eliminate potential confounds due to asymmetries in stimulus-driven arousal [39], (Methods).

To our knowledge, this is the first study to integrate EBR and pupillometry measures to assess how pupil responses relate to value-based learning. Our approach allowed us to characterize the utility of pupil-size recordings for the online tracking of value-based learning. Furthermore, our findings advance the understanding of the interaction between arousal and value-based processes, relevant for studies into Parkinson’s disease.

## Materials & methods

### Participants

In total, 32 naive participants with normal to corrected to normal vision completed the experiment (19 females; mean age = 22.9; age range = 18–32 years). All participants were paid for participation (24€, SD = 1.7€). Two participants were excluded from analyses: one due to excessive blinking and one due to poor task performance (<50% correctly detected reversals), leaving in total 30 participants for analysis. The study was approved by the ethics committee of the Vrije Universiteit Amsterdam and written informed consent was obtained from all participants.

### Task & procedure

Participants were seated in a dimly lit, silent room with their head positioned on a chin rest, 60 centimeters away from the computer screen. They performed a Pavlovian probabilistic reversal learning task where the probability of receiving reward and loss was manipulated. As can be seen in Fig 1a, participants continuously fixated on a central white fixation dot. After 500ms, the fixation dot turned either yellow or purple as a cue for 1000ms, after which the fixation dot changed back to white. After a random interval, drawn from a Gaussian distribution with a mean of 3000ms (standard deviation 300ms) the outcome was indicated by a cash tone (500ms) indicating reward or a white noise tone (500ms) indicating loss. Inter-trial intervals were drawn from a Gaussian distribution with a mean of 3000ms (± 300ms).

On each trial, a purple or yellow colored cue signaled an upcoming reward (+0.10€) or loss (-0.05€) with 80% validity (cue-color mapping was randomly determined across subjects at the start of the experiment). Consequently, in 20% of the trials, the cue elicited an unexpected positive (U+) or unexpected negative (U-) outcome when the reward contingency was fully learned. At unpredictable times, the reward contingency would reverse. (Fig 1b). These reversals would occur once, twice or three times within each run of ~8 minutes (mean number of reversals = 1.98(SD = 0.27) occurring after 29(SD = 3.2) trials, range = 15–59 trials). Participants’ task was to detect reversals in the reward contingency with a keypress.

Prior to the experiment, participants were informed about the range of reversals that could occur on a run. They practiced one experimental run to verify that the purpose of the experiment was clear and they could detect reversals. Each participant performed between 7 and 10 runs of ~80 trials each (mean number of runs = 9.3(SD = 0.9); mean number of trials per run = 78(SD = 4.6)) with small breaks in-between runs. After each experimental run, feedback was provided about the number of correctly detected reversals that run. There was no performance incentive other than the feedback provided about reversal detection performance.

### Stimuli

Stimuli were presented on a 21-inch Iiyama Vision Master 505 MS103DT with a spatial resolution of 1024 x 768 pixels, at a refresh rate of 120Hz, with mean luminance 60 cd/m2. All experiments and data analysis were performed using custom software written in Python, using the Visionegg (v1.2.1), Numpy (v1.11.2), Scipy (v0.18.1), FIRDeconvolution (v0.02), Hedfpy (v0.0dev1), PyPsignifit (v3.0) and MNE (v0.14) packages. The effect of light on pupil responses was minimized by keeping the background luminance of the display constant throughout the experiment. For similar reasons, the two visually presented stimuli -a purple and yellow colored dot- were of small size (r = 0.2°). Prior to the main experiment, we equated subjective tone saliency of the reward and loss tone per participant using a two-interval forced choice (2-IFC) experiment. On each trial, the loss and the reward tone were played with a 1s. inter-stimulus interval, and the participant judged which of two tones was more salient. We varied the intensity of the loss tone according to the method of constant stimuli [40], and fitted a psychometric curve (cumulative Gaussian function) to the participant’s reports using the PyPsignifit package [41]. The point of subjective equisalience of the sounds is the 50% point of this curve, and this intensity value was used for the loss tone in the main experiment. Additionally, the impact of monetary rewards and losses was equated according to prospect theory (a factor of 2, [42]). In our experiment, this translated to per-trial rewards and losses of +0.10€ and -0.05€, respectively.

### Eye blink rate recordings

We recorded spontaneous EBR throughout the experiment and quantified blink rate from the entire pupil size time series. This resulted in ~80 minutes of EBR estimation data per participant, which allows for robust estimation of this trait-like variable [43–47]. Although other procedures have been described to measure spontaneous EBR [31,48], measuring EBR from continuous eye tracking data is reliable [49]. EBR has been reported to have high measurement reliability across assessments [46], suggesting it can be used to reliably index individual striatal dopaminergic tone. Because spontaneous EBR is reported to be stable only during daytime [50], data was collected before 6 P.M. Furthermore, participants were asked to sleep sufficiently the night before the recording and to avoid the use of alcohol and other drugs of abuse.

## Data analysis

### Behavioral data

For all runs, we recorded the timing of the behavioral report that indicated the participant's detection of a reversed state. At the start of each run, the participant had to learn the reward contingency and had to press the spacebar when a change was detected in the learned reward contingency. We used the timings of the experimental reversals to determine the number of reversal blocks within a run, where each experimental reversal marked the start of new reversal block. We categorized the timing of each behavioral report according to signal detection theory [40]. A hit was defined as the first behavioral report occurring after the experimental reversal. A miss was defined as the absence of a behavioral report after an experimental reversal. Then, the hit rate was calculated as the proportion of experimental reversals to which participants detected the reversal. A false alarm was defined as a behavioral report occurring before the first experimental reversal or occurring after the first behavioral report (a hit) within a reversal block. Because participants were only asked to detect the presence of a reversal, but not the absence of it, correct rejections remained undefined. Thus, the false alarm rate was calculated as the proportion of incorrectly reported reversals relative to the total number of reversal blocks. Detection times were quantified as the median number of trials from the experimental reversal to "hit" behavioral reports. Detection time variability was quantified as the standard deviation of the number of trials from experimental reversal to "hit" behavioral reports.

**Fig 2:**
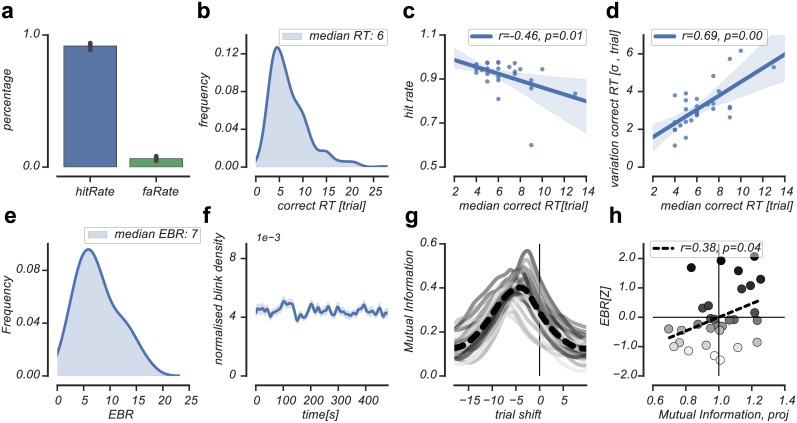
Behavioral and EBR data. (A): Average hit rate and false alarm rate across subjects (B): Response time distribution of correctly detected reversals across subjects. (C): Relation between the hit rate and correct reversal detection time. (D): Relation between correct reversal detection time and detection time variability. (E): EBR distribution across subjects. (F): Blink density across a run of 8 minutes averaged across subjects. (G): Mutual information (MI) between experimental and reported reversals plotted as a function of time-shifts in the reported reversals. Across subjects, shifting the reported reversals 5 trials back towards the experimental reversal (at trial shift = 0) resulted in the highest MI estimate (dashed line). Time units on x-axis are trials. Solid lines reflect individual MI estimates, colored by individual EBR rank (low EBR = light gray, high EBR = dark gray). (H): Individual MI estimates projected on the average MI estimate were positively related to EBR, indicating that individuals who detected reversals more consistently across runs had relatively higher EBR (low EBR = light gray, high EBR = dark gray).

We quantified participants' response consistency in detecting reward contingency reversals by calculating the mutual information (MI) between the experimental and reported reversals. The experimental and reported reversals were two sequences consisting of zeros and ones that marked the objective and subjective states of the world, respectively. MI quantifies the amount of information obtained from one random variable through the other random variable. As such, it measures the relation between two variables, but is not sensitive to the sign of this relation. This is important in our analysis, as participants reported only changes in the state of the world and not the state of the world itself, rendering the sign of the reported state of the world ambiguous. For each participant and run, we calculated the MI between experimental and reported reversals as a function of time-shifts of the reported reversals. This resulted in a per-participant curve that quantifies MI between experimental and reported reversals for a range of time-shifts in the reported reversals (Fig 2g). Higher MI estimates reflected more consistent reversal detection performance across runs. We projected the individual MI curves onto the averaged MI curve to get a scalar value of participants' relative reversal detection response consistency. Additionally, we used the location of the peak of the average MI curve -reflecting the optimal number of time-shifts of the reported reversals relative to the experimental reversals- to validate our quantification of average reversal detection times obtained via the categorization approach.

### Preprocessing of eye-tracking data

The diameter of the left eye’s pupil was recorded at a 1000Hz using an EyeLink 1000 Tower Mount (SR Research). Blinks and saccades were detected using standard EyeLink software with default settings and Hedfpy, a Python package for preprocessing pupil size data. Periods of data loss during blinks were removed by linear interpolation, using an interpolation time window of 200ms before until 200ms after a blink. Blinks not identified by the manufacturer’s software were removed by linear interpolation around peaks in the rate of change of pupil size, using the same interpolation time window.

As illustrated in Fig 1c, the interpolated pupil signal was filtered into transient and tonic pupil signals. We defined pupil responses as changes in phasic or tonic pupil size that could either be dilations (e.g. due to feedback presentation) or constrictions (e.g. following increases in luminance due to cue presentation). To analyze transient pupil responses, the pupil signal was band-pass filtered between 0.03Hz and 4Hz, using third-order Butterworth filters. Low-pass filtering of the transient pupil signal removed measurement noise that did not originate from a physiological source, as the system controlling pupil size responds in a sluggish fashion to fast neural inputs [51]. High-pass filtering the transient pupil signal allowed us to perform independent analyses on transient and tonic pupil responses. Furthermore, to analyze tonic pupil responses, the pupil signal was low-pass filtered with a low-pass cutoff of 0.03Hz. After filtering, tonic and transient pupil data were z-scored per run and resampled to 10Hz.

Blinks and saccades have strong and relatively long-lasting effects on transient pupil responses [52,53]. To remove these influences from the data, blink and saccade regressors were created by convolving all blink and saccade events with their standard Impulse Response Function (IRF) [51,53,54]. These convolved regressors were used to estimate their responses in a General Linear Model (GLM), after which we used the residuals of this GLM for further analysis.

### Event-related analysis of transient pupil responses

Data were divided into epochs based on the condition types in the experiment. For each trial, we calculated the baseline pupil diameter in the time window of fixation (-0.5s. until 0s. to cue onset) and subtracted this baseline value from the pupil time course of the same trial, after which the data epochs were averaged. For the cue interval, the two condition types were: loss cue (L) and reward cue (R), while collapsing over cue colors. For the outcome interval, the four condition types were: loss cue, loss outcome (LL); reward cue, loss outcome (RL); reward cue, reward outcome (RR); and loss cue, reward outcome (LR). Pupil responses after unexpected reward (U+) were calculated by subtracting the pupil response to expected reward trials (RR) from the pupil response to unexpected reward trials (LR). Pupil responses after unexpected loss (U-) were calculated by subtracting the pupil response to expected loss trials (LL) from pupil responses to unexpected loss trials (RL). This method corrected for any feedback tone-specific changes in pupil dilation.

We selected the latter half of trials of each reversal block (mean = 14.5(SD = 1.6) trials per block, total number of selected trials per participant = 361.5(SD = 35.6)) to ensure the analyses of transient pupil responses were focused on those trials where participants had fully learned the reward contingency, resulting in maximal transient task-related pupil responses (Fig 1b).

### Deconvolution of tonic pupil responses

Tonic pupil responses were analyzed using FIRDeconvolution, a Python package which performs Finite Impulse Response fitting [55]. A previous study investigating tonic pupil signals reported a systematic decay in baseline pupil size that unfolded on a timescale of minutes [53]. To account for systematic decay in baseline pupil diameter size over time, we pre-processed the tonic pupil signal by estimating and removing pupil diameter drift in each run using an exponential decay function:

$$y=ae^{-tx}+b$$ (1)

where parameters *a*, *t* and *b* were estimated per subject. Here, *a* is the gain/amplification factor, *t* is a time constant that describes how rapidly pupil size decreases over time, *x* is each run’s pupil data and *b* is the offset of pupil size on the y-axis. Next, we applied zero-padding to each run to avoid edge-artefacts in situations where the deconvolution interval of 120 seconds would exceed a run’s sample limits. Tonic pupil time courses were estimated in the interval of 60 seconds before until 60 after the following 2 event types: perception of a reversal (as indicated by the behavioral report) and the true experimental reversal point. Next, tonic pupil responses were deconvolved using ordinary least squares (OLS) regression:

$$h={\left(X^{T}X\right)}^{-1}X^{T}y$$ (2)

where *y* is the pupil signal time series and *X* is the design matrix consisting of a set of vectors that contain ones at all sample times relative to the event timings of which we want to estimate the pupil response, and zeros elsewhere. *h* then contains the resulting deconvolved pupil responses of all separate event types. Design matrix *X* had 2400 columns (-60s. to 60s. at 10Hz, 1200 samples per event type and 2 event types per deconvolution operation).

### Eye blink rate & eye blink density calculations

EBR per minute was quantified from the complete pupil size time series recordings. This resulted in a per-participant scalar EBR estimate based on ~80 minutes of recordings. Because there is evidence, albeit mixed, for sex differences in EBR [45,56], we normalized EBR based on sex. Because blinks have a strong and prolonged effect on pupil size, we controlled for the possibility that differences in the number of blinks over time between condition types or individuals would affect pupil size differently. To do so, we calculated blink density over time using kernel density estimates for both the transient and tonic pupil data.

To estimate the relation between blink density and transient pupil responses, blink events were convolved with a standard, one-dimensional Gaussian function (kernel SD = 2s.) and normalized per participant by dividing the convolved blink events by the total number of blink events. The convolved, normalized blink events where divided into epochs and averaged per experimental condition type. To estimate the relation between blink density and tonic pupil responses, blink events were convolved with a one-dimensional Gaussian function (kernel SD = 20s.) and normalized. The convolved, normalized blink events were used as the input signal in a deconvolution analysis similar as described above, with reversal detection (behavioral report) as event type.

Furthermore, we investigated the stationarity of blink events within a run to exclude the possibility that transient pupil responses were affected by systematic drift in the occurrence of blinks. Across subjects, we calculated the average blink density of a run using transient kernel density estimates (kernel SD = 2s.) and tested signal stationarity using the Augmented Dicky-fuller test [57].

### Statistical comparisons

We used nonparametric permutation cluster-based t-tests [58] -as implemented in the MNE package [59,60]- to correct for multiple comparisons and to test for significant differences between time series signals to condition types as well as their difference to baseline. Specifically, for a time series signal we calculated the cluster-based t-test statistic describing whether the observed mean significantly deviated from zero. Then, we permuted the time series signal by generating random sign flips of the samples and calculated the corresponding cluster-based t-test statistic. This procedure was repeated 1024 times, resulting in a histogram of random cluster-based t-test statistics. The p-value (corrected for multiple comparisons) was obtained by calculating the proportion of random cluster-based t-test statistics that resulted in a larger test statistic than the observed one.

Correlations across time between EBR and time series signals were calculated using bootstraps [61]. Here, we randomly drew with replacement 1000 new EBR and time series value pairs and correlated them for each time point. From the resulting bootstraps, 95% confidence intervals and p-values were calculated based on a two-sided hypothesis test, where the p-value was the fraction of the bootstrap distribution that fell below (or above) 0.

A nonparametric permutation cluster-based correlation test [62] was performed to correct for multiple comparisons and to test for significant correlations between EBR and time series signals. Specifically, we calculated the cluster-based t-test statistic corresponding to the cluster of time points where the time series signal significantly correlated with EBR. Next, we randomly permuted EBR values (N = 30) across subjects and calculated the corresponding cluster-based t-test statistic. This procedure was repeated 1000 times, resulting in a histogram of random cluster-based t-test statistics. Corrected p-values were obtained by calculating the proportion of random cluster-based t-test statistics that resulted in a larger test statistic than the observed one.

## Results

### Behavior

Reversal detection performance was high (mean hit rate = 91.7%(SD = 7.3%), range = 60%-97.7%, Fig 2a). On average, correctly detected reversals were detected after 6.4(SD = 3.2) trials (range = 1–27 trials, Fig 2b). As expected, higher hit rates were related to faster reversal detection times (Pearson's r(28) = -0.46, p = .01, Fig 2c) and less detection time variability (Pearson's r(28) = 0.69, p < .01, Fig 2d). This pattern of findings was additionally supported by the MI method, indicating that experimental and reported reversals shared the highest amount of variance when the reported reversals where shifted 5 trials back in time (Fig 2g). This suggested an average reversal detection time of 5 trials, which was close to the average reversal detection times (6 trials) obtained via the categorization approach (see Methods).

On average, participants blinked 7.6(SD = 3.88) times per minute (range 1.8–16.9 Fig 2e), an average that was lower than reported by other studies [31,48,63]. Participants with low EBR blinked 4.5(SD = 1.5) times per minute (range 1.8–7.0), whereas participants with high EBR blinked 10.9(SD = 2.9) times per minute (range 7.3–16.9). As reported previously [45], female participants blinked more often than male participants (t(29) = 2.24, p = .03, independent-samples t-test; females: 8.8(SD = 3.8), range 2.14–16.9; males: 5.7(SD = 1.8), range 1.8–12.2). We corrected for this by normalizing EBR by sex and used the normalized EBR values in all subsequent analyses. The general pattern of main findings remained the same when using non-normalized EBR. The occurrence of blinks within a run (of approximately 8 minutes) was constant, as we could reject the null hypothesis of non-stationarity in blink density across subjects (ADF = -11.7, p < .001, Augmented Dickey-Fuller Test; Fig 2f). Behaviorally, individuals with relatively high EBR reported reversals more consistently across runs, as was indicated by a positive correlation between EBR and individual mutual information (MI) estimates, projected onto the average MI estimate (Pearson's r(28) = 0.38, p = .04, Fig 2h). For the depiction of subsequent results that pertain to the relation between pupil responses and EBR, we performed a median split of participants according to their EBR.

### Identical arousal-based pupil responses during value expectation and outcome evaluation correlate differently with EBR

**Fig 3:**
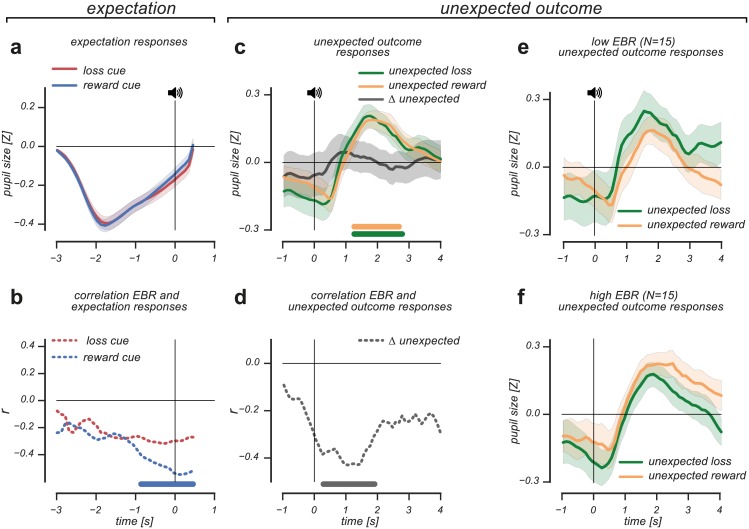
Arousal-based pupil responses during value expectation and outcome evaluation show different correlation patterns with EBR. (A): Reward and loss expectation elicited identical pupil responses. (B): Reward expectation correlated significantly with EBR, indicating that reward expectation elicits stronger pupil dilation in individuals with low compared to high EBR. (C): Violated reward and loss expectations resulted in unsigned, transient pupil dilation. No dilation differences were observed between U- and U+ responses, which was reflected in the ΔU response. (D): ΔU correlated significantly with EBR, indicating that the variability in the ΔU response related to individual differences in EBR (E & F): A median split on individual differences in EBR visualizes the correlation between ΔU and EBR, where pupil dilation response patterns to unpredicted outcomes reversed for individuals with low compared to high EBR. Horizontal significance designators indicate time points where p<0.05. Error bars are standard error of the mean. Statistics based on cluster-based (correlation) permutation tests, n = 1000.

To understand the relation between value-based processes -alongside those related to arousal- and pupil size, we first investigated transient pupil responses during the expectation of value and their relation to EBR. Across subjects, the expectation of upcoming reward and loss caused identical pupil responses (Fig 3a). The pupil initially constricted due to cue presentation, after which it gradually dilated until the presentation of the outcome. As we observed no difference in the gradual dilation pattern between reward and loss expectation events, this suggests that the pupil reflects arousal caused by uncertainty about the upcoming outcome. Interestingly, when we the correlated the observed transient pupil responses with individual differences in EBR, we found that EBR was negatively correlated with the transient pupil response during reward expectation (cluster p-value < .001, 0.8s. pre-event until 0.4s. post-event, nonparametric permutation cluster-based correlation test; Fig 3b). This indicated that the pupil of individuals with relatively low EBR dilated more strongly when they were expecting reward; an unexpected finding given the generally observed positive relationship between EBR and striatal dopamine levels [20–27].

Next, we investigated the transient pupil response during the receipt of unexpected loss (U-) and unexpected reward (U+), as these events allowed us to further differentiate between the impact of arousal- and value-based signals. In this experiment, we matched subjective sensory and incentive impact of outcome events, thus avoiding systematic differences in stimulus-driven arousal between outcome events.

We observed that both unexpected loss (U-) and unexpected reward (U+) caused transient pupil dilation, (U- cluster p-value = 0.04, 1.2s.-2.7s. post-event, Cohen's d = 0.74; U+ cluster p-value = 0.02, 1.2s. until 2.8s. post-event, Cohen's d = 0.83; non-parametric cluster-based one sample t-test; Fig 3c). There were no response differences between U+ and U- responses, as the difference between these pupil responses (ΔU) never reached significance (all p-values>0.7, BF = 0.2). These findings indicate that across subjects, unexpected outcomes, irrespective of their value, generated pupil responses reflecting surprise [10–13].

While pupil responses elicited by unexpected reward and unexpected loss did not reflect transient value modulations, we further investigated how the observed pupil responses related to individual differences in EBR. To quantify this, we calculated the difference between U+ and U- pupil responses (ΔU, Fig 3c) and evaluated how this differential pupil response (based on violated reward and loss expectations) was related to EBR.

As shown in Fig 3d, the difference between unexpected reward and unexpected loss pupil responses (ΔU) correlated significantly with EBR (cluster p-value = 0.01, 0.2s. until 1.8s. post-event, nonparametric permutation cluster-based correlation test), even though the mean response amplitude difference across participants hovered around zero. This result suggests that across subject variability in the ΔU pupil response related to individual differences in EBR. Furthermore, we observed that the correlation between ΔU and EBR became significant shortly after outcome presentation, suggesting that the effect was partly driven by differences in value expectation that were present in pupil size prior to outcome evaluation. We further visualized the relation between EBR and the ΔU pupil response using a median split on EBR. As shown in Fig 3e & 3f, the relative response amplitudes of U- and U+ responses reversed for individuals with high compared to low EBR. In individuals with relatively low EBR, unexpected loss elicited stronger pupil dilation than unexpected reward, whereas the opposite response pattern was observed in individuals with high EBR.

Overall, we observed arousal-based influences on pupil size both during value expectation and the evaluation of unexpected outcomes. Critically, incorporating individual differences in EBR refined these observations. Increases in pupil size during reward expectation were stronger for individuals with lower compared to higher EBR. Consistent with this finding, violations of reward expectations (unpredicted loss, U-) caused stronger pupil dilation in individuals with lower compared to higher EBR.

### Tonic pupil size tracks the perception of a reward contingency reversal and correlates with EBR

**Fig 4:**
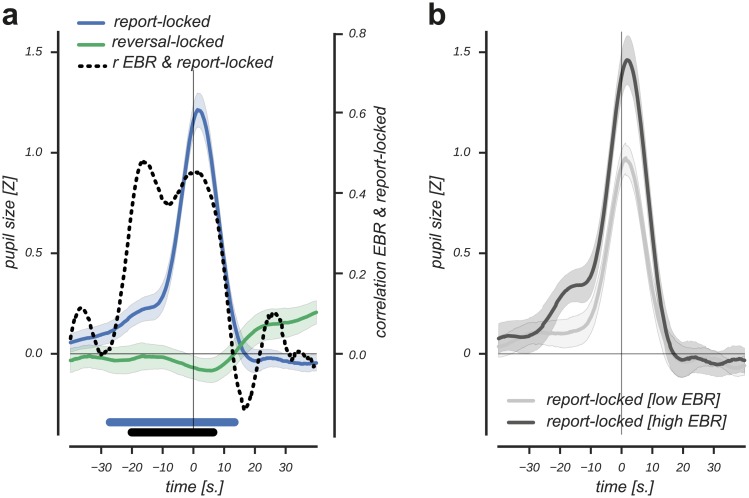
Tonic, report-locked pupil responses track reversal detection and correlate with EBR. (A): Reversal detection was reflected in a tonic, report-locked response starting 27s. prior until 13s. post-event. No significant pupil response was observed at the time of the experimental reversal (both the report and experimental reversal are plotted at t = 0). A positive correlation between the report-locked response and EBR (dashed line) starting 21s. prior until 7s. post-event indicated that individuals with relatively high EBR show stronger reversal detection responses (black solid horizontal significance designator; correlation values correspond to Pearson r values on the right y-axis). (B): Individuals with relatively high EBR showed stronger reversal detection responses that started earlier in time and lasted longer. Error bars are standard error of the mean. Statistics based on cluster-based (correlation) permutation tests, n = 1000.

Next, we investigated how the evidence integration of a reward contingency reversal was reflected in the tonic pupil data (Fig 1c), as we observed earlier that higher reversal detection hit rates predicted faster evidence integration of a reward contingency reversal (Fig 2c). As shown in Fig 4a, reversal detection was clearly visible in the tonic pupil response locked on the behavioral report. This report-locked pupil response started to increase significantly well before the report (cluster p-value < 0.001, 27s. pre-event until 18s. post-event, Cohen's d = 2.6; non-parametric cluster-based one sample t-test). Moreover, the tonic response increase was specific to the detection of a reversal (that was indicated by the behavioral report) and not related to the actual experimental reversal (light green line, Fig 4a). This suggested that the response was specific to participants' perception of and response to the reversed reward contingency. The prolonged duration of the tonic report-locked response precludes that it was related to the motor response per se, as the typical duration of pupil responses triggered by such an event is 2–3 seconds [8,52].

As EBR has been associated with learning from negative outcomes [30,31], we additionally investigated whether the tonic report-locked response, potentially driven by the experience of consecutive violations about the state of the world, related to EBR. As shown in Fig 4a (dashed line), we observed a positive correlation between individual differences in EBR and the tonic report-locked response (cluster p-value = 0.002, 21s. pre-event until 7s. post-event, nonparametric permutation cluster-based correlation test). We visualized the positive correlation by estimating the report-locked tonic pupil response separately based on a median split on EBR. The dark and light grey curves in Fig 4b illustrate that individuals with high EBR showed a relatively stronger report-locked detection response that started earlier in time.

Taken together, we observed that a slow dilatory response tracked participants' detection of a reversal in reward contingency. This pupil response started to rise well before the behavioral report, and did not relate to the actual reversal of the reward contingency. Furthermore, we observed a positive relation between EBR and the tonic report-locked response, indicating stronger reversal detection responses in individuals with higher EBR.

### Blink density control analyses

One could argue that the observed correlations between pupil responses and EBR could be explained by differences in the blink occurrences over time that correlated with EBR, as blinks profoundly modulate pupil size [52,53]. To control for this potential confound, we analyzed blink density patterns during reversal detection, expectation of value, and the evaluation of unexpected outcomes. Furthermore, we correlated the observed blink density patterns with EBR measures over time.

**Fig 5:**
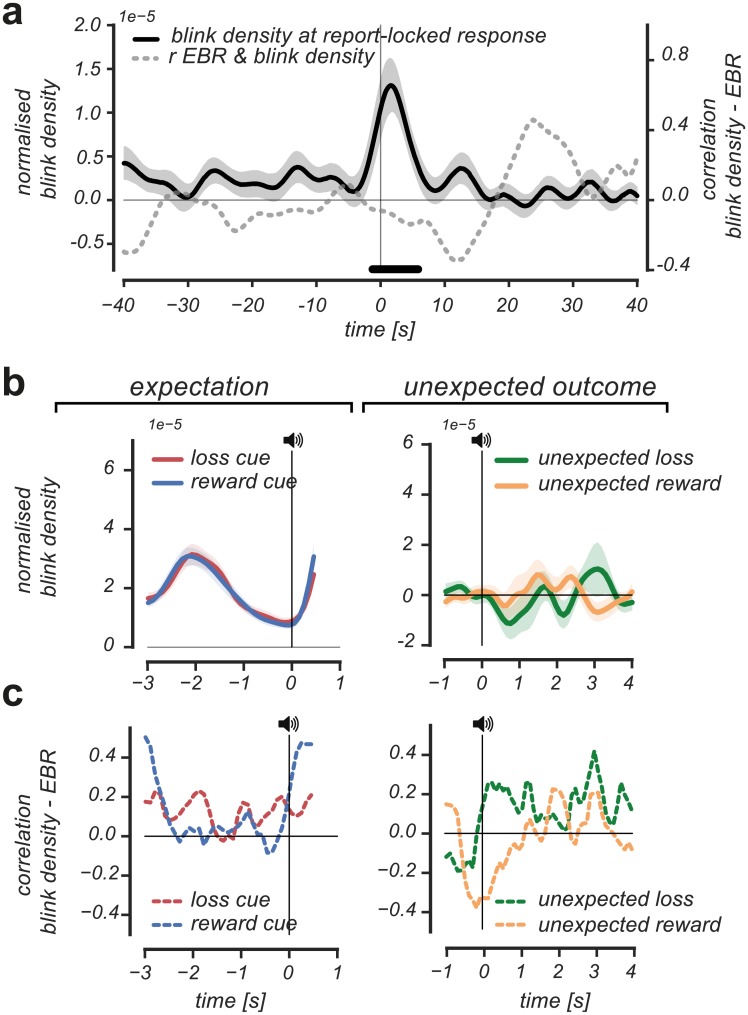
Blink density patterns and their relation to individual differences in EBR. (A): No deviations were observed in blink density patterns prior to the detection of a reversal. At the moment of the behavioral report, blink density significantly increased 2s. pre-event until 6s. post event (black horizontal significance designator). Individual differences in EBR did not correlate with blink density patterns during the interval of reversal detection (grey dotted line, corresponding to the Pearson r values on the right y-axis). (B): No differences in blink density pattern were observed between reward and loss expectation events (left panel) nor between unexpected reward and unexpected loss events (right panel). (C): Blink density patterns did not significantly correlate with individual differences in EBR during the expectation of reward and loss (left panel), nor during the experience of unexpected reward and unexpected loss (right panel). All statistics based on cluster-based (correlation) permutation tests, n = 1000.

There were no significant deviations in blink density patterns prior to reversal detection, arguing against the hypothesis that changed blink density affected the rise in the tonic report-locked response (Fig 5a). At the time of the behavioral report, we did observe a significant increase in blinks (cluster p-value = 0.01; 2s. pre-event until 6s. post-event, non-parametric cluster-based one sample t-test). However, the increase in blinks during reversal detection could not have affected the rise of the tonic report-locked response much, as the response already significantly increased 27 seconds prior to report. Furthermore, there were no significant differences in blink density patterns around reversal detection between individuals with high and low EBR, evidenced by the fact that EBR measures did not correlate with blink density patterns (Fig 5a, correlation values correspond to Pearson *r* values on the right y-axis).

At the fast trial-based time scale we did not find evidence for differences in blink density patterns between reward and loss expectation events (Fig 5b, left panel), nor differences in blink density patterns between unexpected loss and unexpected reward events (Fig 5b, right panel). Furthermore, we did not observe clusters in time where blink density patterns correlated significantly with individual differences in EBR during reward and loss expectation (Fig 5c, left panel), nor during the experience of unexpected reward or unexpected loss (Fig 5c, right panel). This indicates that individuals with high compared to low EBR showed no systematic differences in blink density pattern around the expectation of value or the evaluation of unexpected outcomes.

To conclude, we did not find differences in blink density patterns prior to reversal detection or during the expectation or evaluation of value. Neither did we find that blink density patterns correlated with individual differences in EBR during reversal detection or the expectation or evaluation of value. This argues against the possibility that the observed differences in pupil responses between individuals with high compared to low EBR could be explained by differences in blink density patterns in the described intervals.

## Discussion

We investigated whether pupil responses and EBR could be utilized to track value-based learning in a Pavlovian reversal learning task. When studying pupil responses within trials, we observed arousal-based influences on pupil size both during outcome expectation and the evaluation of unexpected monetary outcomes. We refined these findings by showing that the observed transient pupil responses were related to individual differences in EBR, a behavioral correlate of striatal dopaminergic tone [20–27]. This suggests that transient pupil responses may provide an additional view into cognitive processes related to value-based learning [35–38]. When focusing on the detection of reward contingency reversals across trials, we observed increases in tonic pupil size already several trials prior to the behavioral report. Furthermore, EBR correlated positively with tonic pupil size, indicating stronger tonic reversal detection responses in individuals with higher EBR.

The observation of identical anticipatory pupil responses to upcoming rewards and losses indicated that, averaged across participants, pupil size did not reflect the sign of the expected outcome. This is consistent with findings of similar dilatory pupil responses to most and least preferred stimuli [33,38]. Moreover, our finding of similar transient pupil dilation during the experience of unexpected reward and unexpected loss emphasizes that the underlying cause of the pupil response was surprise, associated with errors in built-up expectations [6,11–13,64].

Additionally, we utilized EBR to investigate more subtle pupil response modulations related to value-based learning, over and above to the observed arousal-based effects on pupil size. We observed that across-subject variability in pupil responses during the expectation and evaluation of value related to EBR. This suggests that the strength of transient pupil dilation indexes individual value sensitivity, which affects how individuals learn from reinforcing feedback [16–19,29]. Again, these results emphasize that pupil responses reflect arousal-based influences, as both the expectation and evaluation of value related to increases in pupil size.

Individuals with relatively low EBR anticipated reward more strongly, as indicated by their stronger pupil size increase. This is a novel finding, as the relation between EBR and reward expectation during Pavlovian reversal learning was not investigated before. Partly related to this finding is a recent study that investigated whether pupil responses indexed motivational preparation for upcoming reward in Parkinson's patients on and off their dopaminergic medication [14]. They found that dopaminergic medication reinstated blunted pupil responses during saccade preparation to earn reward, where higher reward incentive elicited stronger pupil dilation than lower or no reward incentive. However, it remains difficult to compare our findings with those of Manohar et al. (2015) as there have been clear pupillary abnormalities observed in patients suffering from Parkinson's disease [65,66].

When reward expectations were violated, individuals with lower EBR showed a stronger pupil response to the experienced unexpected loss. These findings show some consistency with studies observing a link between EBR and the outcome of reinforcement learning processes [30,31]. Cavanagh et al. (2014) observed that decreasing striatal dopamine levels, as indexed by EBR, led to increased punishment avoidance after response conflict [30]. Moreover, Slagter et al. (2015) showed that lower EBR predicted a value-based choice strategy focused on avoiding negative outcomes [31]. These studies suggest that low EBR relates to learning from negative reinforcements, which parallels our observation of a relation between lower EBR and stronger pupil dilation after unexpected loss. However, we also observed that higher EBR related to stronger pupil dilation after unexpected reward, which is not consistent with Slagter et al. (2015), where no relation was found between high EBR and a value-based choice strategy focused on obtaining reward.

Yet, there are many differences between our experimental design and that of Cavanagh et al. (2014) and Slagter et al. (2015) that preclude any direct comparisons. First of all, our study focused on the relation between pupil responses and EBR during ongoing value-based learning, whereas the aforementioned studies focused on value-based choice strategies that were learned during reinforcement learning. EBR may relate differently to learned value-based choice strategies and instantaneous value experiences, necessitating future experimental work that differentiates between these two processes. Pupillometry and EBR measures combined with detailed behavioral data might provide tools to accomplish this. A further difference can be found in our employment of Pavlovian conditioning, whereas both Cavanagh et al. (2014) and Slagter et al. (2015) employed operant conditioning that involved behavioral choice. Thus, EBR may also relate differently to Pavlovian and operant conditioning mechanisms [67,68].

In the evaluation of tonic pupil responses associated with reversal detection, we observed strong increases in pupil size. Tonic pupil size, locked to the behavioral report of a reversal, increased several trials prior to the actual report, suggesting that it indexed participants' uncertainty about the encounter of a potential contingency reversal. This finding relates to studies showing that increases in per-trial baseline pupil diameter predicted exploratory decision making [5], task disengagement [4], and uncertainty about the underlying task contingency [6]. Fluctuations in baseline pupil diameter are thought to be mediated by arousal-based signals originating from the locus coeruleus-noradrenaline (LC-NE) system [1], and are observed to correlate with tonic LC activity [1,69] and LC BOLD responses [70]. Thus, the observed tonic reversal detection response might index the build-up of uncertainty or the need to update current beliefs, energized by increases in tonic LC activity. This functional role of tonic LC activity was observed in an electrophysiological study in nonhuman primates, where a rise in tonic LC activity was observed immediately after a reversal in task contingency, but long before the adaptation of the monkey's behavior [71]. Recently, a more specific hypothesis was proposed about the role of tonic NE in the orbitofrontal cortex during reversal learning [72]. Here, reversal detection was hypothesized to lead to a rise of tonic NE to evoke the discarding of old cue-outcome associations, where after tonic NE levels would drop to allow stabilization of the newly acquired contingency. Our results are in line with this hypothesis and suggest that tonic pupil size tracks the unfolding of decision-making during reversal detection.

Apart from the potential arousal-based influences on tonic pupil size during reversal detection, we additionally observed that the tonic report-locked response correlated with EBR. This suggests that variability in the amplitude of the reversal detection response might reflect EBR-indexed individual differences in striatal dopaminergic tone. While our data do not allow us to draw inferences about neuromodulatory brain processes, our findings allude to the described role of dopamine (DA) in controlling flexible behavior [73,74]. DA is thought to gate the signal that triggers state updating in frontal cortex via modulations of the decision threshold in the basal ganglia [75–77], where higher DA levels facilitate flexible state updating by reducing the threshold to respond [75]. The positive correlation pattern between EBR and the tonic reversal detection response might relate to this mechanism, where stronger detection responses in individuals with high EBR possibly reflect higher arousal or sensitivity to state updating after detecting environmental change.

Our data support the current theory that arousal-based mechanisms shape pupil responses [1,5,78]. We extend current insights by showing that the simultaneous analysis of EBR and pupillometry measurements provide an additional view into processes related to value-based learning. That is, across-subject variability in pupil dilations during value expectation and evaluation relate to individual differences in EBR-indexed striatal dopaminergic tone (but see [79]).

Although the effects reported here are based on a young and healthy sample of the population, our data and that of other recent studies [14,15,80] suggest pupil responses might be of use to index reward learning processes in clinical settings, for example to study impaired reward-based learning in Parkinson’s disease (PD). PD patients often suffer from disorders in movement and executive functioning [81,82], that renders behavioral responses in some occasions difficult to use. Indexing value-based learning indirectly via physiological measures like pupil size reduces strong behavioral requirements on patients, making pupillometry a potentially promising measure to access value-based learning.
